# Cholera in Granite Regions

**Published:** 1849-01

**Authors:** 


*Cholera in Granite Regions.*—Dr. C. T. Jackson, of Boston, distinguished for his scientific acquirements, publishes recently an opinion that the cholera will not fasten upon New England, or rather in this section of the country, as the disease never has been very destructive in granite regions. His arguments are philosophical; yet it will be recolected that the disease once appeared here, and the apprehensions of its second appearance, to an equal extent, at least, are well grounded. According to Dr. Jackson's views, in those sections where lime is found, the cholera has invariably exhibited its most deadly activity, and it is in such regions alone that the greatest danger in this country is to be apprehended.

Although the municipal authorities seem to be making some preparation for the reception of the pestilence at different Atlantic points, all experience proves that those defensive measures, which relate only to importation of the disease, are utterly worthless. Cholera cannot be kept at bay, or turned from its course. Neither does dieting in any particular manner render the individual less liable to contract the disease, or lessen its violence when developed, if credit is to be placed in the history of this remarkable pestilence. But this circumstance should not operate to prevent public bodies and municipalities from abating nuisances, and bettering the condition of the poor in those plague spots which are found in every city, and town of magnitude, in which a certain class of people invariably establish themselves.—*Boston Medical and Surgical Journal.*

